# Tregs and persistent *P. gingivalis* colonisation: a plausible mechanism for cardiovascular risk in edentulism

**DOI:** 10.3389/froh.2026.1895121

**Published:** 2026-09-14

**Authors:** Jelena Roganović

**Affiliations:** Faculty of Dental Medicine, Department of Pharmacology in Dentistry, University of Belgrade, Belgrade, Serbia

**Keywords:** cardiovascular disease, edentulism, periodontitis, *porphyromonas gingivalis* persisters, regulatory t cells

## Abstract

Poor oral health is commonly linked to cardiovascular disease through the established association between periodontitis and systemic inflammation. This relationship is usually explained by bacterial dissemination from periodontal pockets, lipid dysregulation, and oxidative stress, all of which may contribute to endothelial dysfunction and atherothrombotic changes. However, this conventional framework leaves an important question unresolved: if the main infectious and inflammatory niches are removed after complete tooth loss (edentulism), how does edentulism remain associated with increased cardiovascular risk? Epidemiological studies consistently show that edentulous individuals continue to have elevated cardiovascular morbidity and mortality, yet mechanistic explanations for this association remain underdeveloped. Therefore, this paper proposes a plausible biological model to explain persistent cardiovascular risk in edentulism. The model argues that, after tooth loss, the oral environment is not biologically inert but rather reorganised into a distinct ecological and immunological niche shaped by dentures, altered salivary flow, nutrient changes, microtrauma, and residual soft-tissue biofilms. The tongue, oral mucosa, saliva, and denture surfaces may support persistent colonisation by *Porphyromonas gingivalis* and other organisms within distinct soft-tissue and prosthesis-associated biofilms. The model further proposes that local ecological stress, altered nutrient availability, and cytokine changes may favour regulatory T-cell-associated immune tolerance and impair antimicrobial clearance. Microbial products and outer membrane vesicles may provide additional routes of host signalling, although persister-specific OMV production and its vascular relevance in edentulism remain unconfirmed. Persistent cardiovascular risk after tooth loss is likely multifactorial, and the proposed microbial–immune mechanism represents a complementary framework for future longitudinal, microbiological, immunological, and interventional research.

## 1 Introduction

Poor oral health, particularly periodontitis, has long been associated with an increased risk of cardiovascular disease. This relationship has traditionally been interpreted through the combined effects of chronic inflammation and systemic bacterial dissemination (Figure 1). Severe periodontitis is significantly and independently associated with both all-cause and cardiovascular mortality across different populations (1, 2). Teeth provide non-shedding surfaces that allow the accumulation of complex and persistent subgingival biofilms, which are difficult to eliminate. As a result, bacterial products and host immune responses can maintain a chronic low-grade systemic inflammatory state.

**Figure 1:**
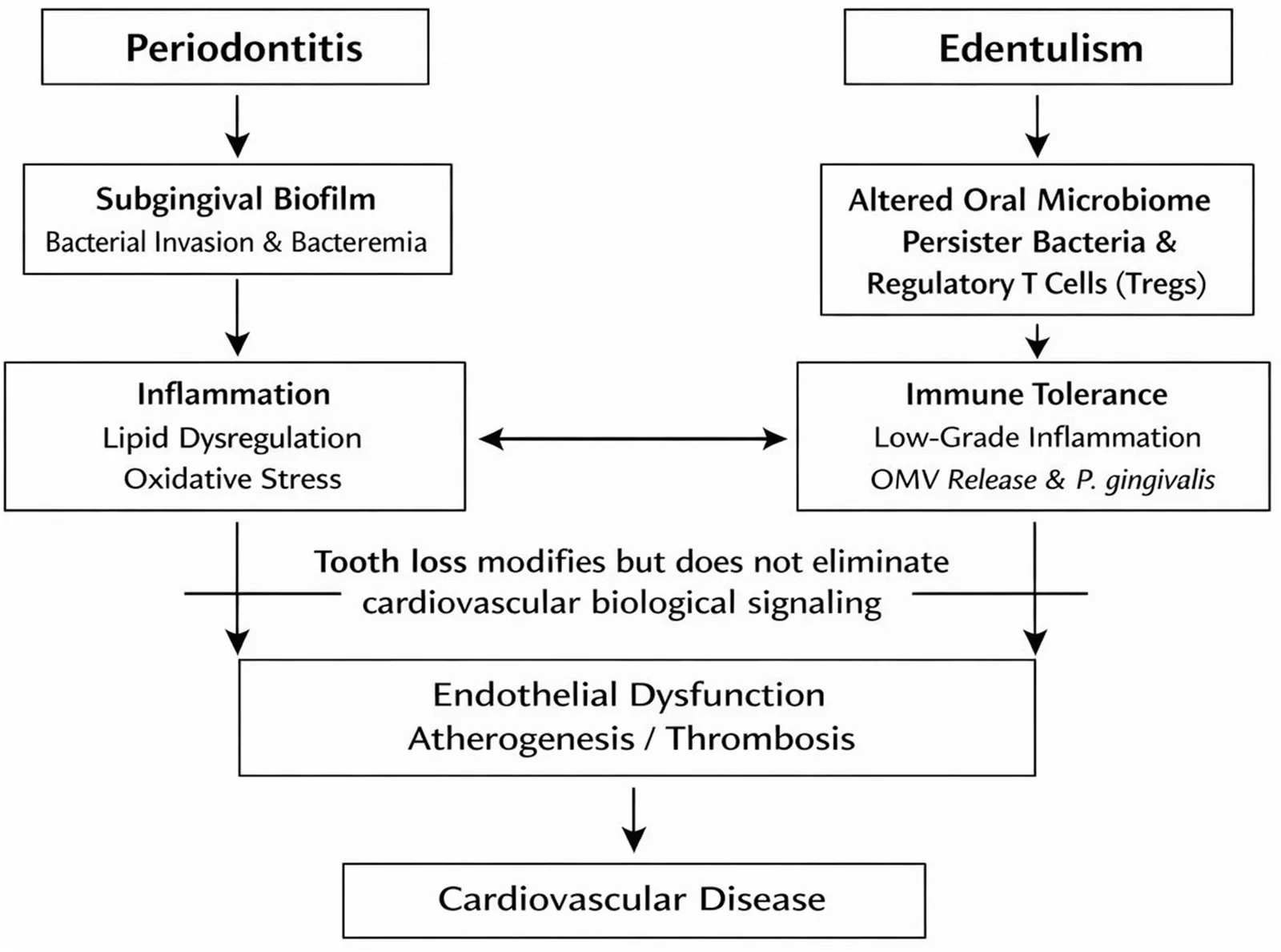
Proposed integrated model showing two biologically distinct but converging pathways linking oral conditions to cardiovascular disease. In periodontitis, persistent subgingival biofilm on tooth surfaces enables bacterial invasion and recurrent bacteremia, leading to systemic inflammation, lipid dysregulation, and oxidative stress. These processes promote endothelial dysfunction, atherogenesis, and thrombosis through increased pro-inflammatory cytokine production, altered lipid profiles, and reactive oxygen species generation. In edentulism, tooth loss removes periodontal pockets but simultaneously creates a modified oral ecological environment characterised by altered microbiota, denture-associated stress, reduced salivary flow, and changed nutrient conditions. Under these conditions, persister forms of oral bacteria such as *P. gingivalis*, together with regulatory T-cell (Treg)-dominant immune responses, may support immune tolerance, low-grade inflammation, and outer membrane vesicle (OMV)-mediated signalling. Although the inflammatory profile differs from active periodontitis, both pathways converge at the level of endothelial dysfunction and vascular injury, suggesting that tooth loss may modify, but not fully eliminate, oral-systemic cardiovascular biological signalling.

In particular, periodontal pathogens such as *Porphyromonas gingivalis, Tannerella forsythia,* and *Treponema denticola* are thought to contribute directly to systemic inflammation by entering the bloodstream during chewing or dental procedures (3). This bacteremia may trigger inflammatory cascades capable of affecting distant tissues, including the vascular system. Molecular evidence further supports this link, as DNA, RNA, and antigens derived from oral bacteria have been detected in atherothrombotic tissues. Studies have shown a higher probability of positive associations between the presence of these bacteria in atherothrombotic tissues and in other sample sources, such as subgingival plaque and serum, in periodontitis patients (4, 5). Experimental models have also demonstrated that *P. gingivalis* fimbriae facilitate bacterial entry into host cells and contribute to the development of atherothrombotic lesions (6). In addition, *in vitro* studies show that bacterial strains expressing *P. gingivalis* hemagglutinin A have an enhanced ability to adhere to and invade human coronary artery endothelial cells (7).

Inflammatory responses further amplify this systemic connection. Peripheral neutrophils from periodontitis patients produce higher levels of pro-inflammatory cytokines, including IL-1β, IL-6, IL-8, and tumour necrosis factor (TNF)-*α*, when stimulated by periodontal pathogens (8). Alterations in lipid metabolism have also been reported in relation to periodontitis. These include changes in total cholesterol, low-density lipoprotein cholesterol, high-density lipoprotein cholesterol, triglycerides, apolipoproteins, oxidised lipids, and lipid-derived inflammatory mediators. Importantly, these lipid abnormalities tend to improve after periodontal therapy (9–11).

Oxidative stress represents another relevant mechanistic pathway. Increased reactive oxygen species accumulation during periodontitis leads to oxidative stress (12, 13). Antioxidant levels in serum and gingival crevicular fluid are also reduced in periodontitis patients, further indicating enhanced oxidative stress (14). This is reinforced by findings from endarterectomy samples, which demonstrate ROS-related neutrophil activation, including the presence of myeloperoxidase (MPO), cell-free DNA, and DNA-MPO complexes (15).

Overall, the interaction between periodontal infection, systemic inflammation, lipid dysregulation, and oxidative stress provides strong mechanistic support for the link between periodontitis and cardiovascular disease. However, this conventional framework leaves an important question unresolved: if the main infectious and inflammatory niches are removed after tooth loss, how does complete tooth loss (edentulism) remain associated with increased cardiovascular risk? This question is clinically relevant since edentulism increases markedly with age, while remaining significantly associated with major cardiovascular conditions, including myocardial infarction, coronary heart disease, congestive heart failure, and stroke (16).

## 2 Edentulism and residual cardiovascular risk

Edentulism, or the loss of all natural teeth, is often considered the end stage of periodontal disease. In principle, edentulism should eliminate periodontitis because there are no remaining teeth or periodontal pockets in which disease can persist. On that basis, one might expect a reduction in systemic inflammation and associated cardiovascular risk. However, multiple studies suggest that edentulous individuals continue to exhibit elevated cardiovascular risk even in the absence of active periodontitis.

A recent systematic review found a significant association between edentulism or having fewer than ten teeth and increased cardiovascular disease mortality (17). Similarly, a prospective cohort study with 20 years of follow-up showed that edentulism was strongly associated with cardiovascular mortality, whereas periodontitis itself was not (18). Tooth loss has also been linked to incident coronary artery disease, acute myocardial infarction, diabetes, and all-cause mortality (19). In addition, it is associated with several cardiovascular risk factors, including elevated C-reactive protein, low HDL cholesterol, and high triglycerides (19).

Thus, removal of local inflammatory foci by tooth extraction does not normalise cardiovascular risk. Supporting this view, a study including three populations with an 11-year follow-up and over twelve thousand participants showed that missing teeth are associated with adverse circulating metabolic markers related to systemic inflammation (20). One widely discussed explanation is that impaired dentition leads to poor nutritional intake, which in turn contributes to increased cardiovascular mortality (21–23), although a systematic review found only a weak association between tooth loss and nutritional status (24).

Other authors have argued that the persistent inflammatory burden may reflect intrinsic host factors rather than periodontitis itself, including pro-inflammatory genetic traits and epigenetic modifications common to both advanced periodontal disease and coronary heart disease (25, 26). It is also plausible that long-term exposure to chronic oral inflammation may initiate secondary pathological responses at distant sites, such as atherosclerotic lesions in blood vessels, which then maintain systemic inflammatory activity even after tooth loss.

However, recent molecular evidence indicated that periodontal pathogens can persist in edentulous mouths (27) suggesting that complete tooth loss may remove periodontal pockets without fully eliminating pathogenic oral microbial reservoirs. This provides a rationale for considering edentulism as a distinct state in which residual soft-tissue and denture-associated biofilms may continue to influence host immunity, vascular stress, and cardiovascular risk. The present model therefore proposes that tooth loss creates a complex edentulous oral microenvironment which may favour low-grade inflammation, tissue stress, altered immune regulation, and persistence of oral pathogens such as *P. gingivalis* explaining why cardiovascular risk can persist despite the removal of tooth-associated periodontal niches. This model offers a new framework for understanding oral-systemic connections after tooth loss and suggests possible targets for future intervention. Although the inflammatory profile differs from active periodontitis, both pathways converge at the level of endothelial dysfunction and vascular injury, suggesting that tooth loss may modify, but not fully eliminate, oral-systemic cardiovascular biological signalling (Figure 1).

A recent synthesis of thirty-two studies concluded that edentulous and denture-associated microbiomes differ from dentate microbiomes, but estimates were highly heterogeneous and influenced by denture characteristics and duration (28). Complete tooth loss causes major ecological reorganisation rather than preserving the same periodontal microbiome. The dorsum of the tongue and denture surfaces can serve as reservoirs, and periodontal organisms, including *P. gingivalis*, may remain detectable after complete tooth loss (27), however, these communities are not necessarily identical to those found in periodontitis. Edentulous and denture-associated biofilms may include organisms adapted to mucosal or acrylic surfaces, including fungal species and bacterial taxa that are not dominant in periodontal pockets (28). Denture biofilms share some features with tooth-associated biofilms but represent a distinct prosthesis-associated ecosystem, not a recreated periodontal pocket (28). Therefore, residual oral–systemic effects may reflect the activity of a broader reorganised microbial consortium rather than *P. gingivalis* alone.

## 3 Persistent *P. gingivalis* colonisation: distinguishing clinical persistence from the possible contribution of persister cells

Since persistent colonisation and the persister phenotype represent distinct biological concepts, their use throughout this Perspective is defined in Box 1.

BOX I Key terminology used in this Perspective

**Persistence/persistent colonisation**

Continued detection or recovery of a microorganism at an anatomical site over time. This demonstrates sustained colonisation but does not, by itself, establish antibiotic tolerance or a persister phenotype.

**Persister cells**

A phenotypic subpopulation of genetically susceptible bacteria that survives otherwise lethal antibiotic exposure through a reversible, non-heritable state of physiological tolerance, often associated with reduced or altered metabolic activity.

**Use in this Perspective**

Clinical studies support persistent P. gingivalis colonisation in edentulous oral niches, whereas P. gingivalis persister cells have been demonstrated experimentally, including after metronidazole exposure, but not yet directly in edentulous patients. Persister cells are therefore considered a biologically plausible contributor to prolonged bacterial survival and host modulation rather than a synonym for persistent colonisation.

### 3.1 Persistence in soft tissues and denture-associated biofilms

The biological basis of persister formation suggests that environmental stress, nutrient shifts, and biofilm-associated metabolic heterogeneity could favour antibiotic-tolerant bacterial subpopulations (29, 30). However, persistent detection or long-term colonisation is not equivalent to a persister phenotype, which is characterised by transient physiological tolerance in a subpopulation of genetically susceptible cells rather than by prolonged presence at an anatomical site alone (29, 30).

Complete tooth loss substantially modify the oral environment by removing non-shedding tooth surfaces and periodontal pockets while retaining the tongue, oral mucosa, saliva, and, in many patients, prosthetic surfaces capable of supporting biofilms. This ecological change may permit continued colonisation by selected periodontal organisms in alternative niches. Environmental stress, altered nutrient availability, biofilm growth, and antimicrobial exposure are also conditions known experimentally to influence bacterial tolerance and persister-cell formation (29, 30). In addition, the edentulous state often involves dentures, altered salivary flow, and changes in local immune responses, all of which may impose environmental stress. Repeated antimicrobial exposure, whether related to prosthetic maintenance or secondary infections, could further enrich for persister cells that survive treatment while non-persister cells are eliminated. Their low metabolic activity may also enable long-term persistence without provoking strong immune responses. However, whether these conditions generate or enrich *P. gingivalis* persister cells in edentulous patients has not been established although clinical observations support persistence: red-complex organisms, including *P. gingivalis*, have been detected on the dorsum of the tongue in completely edentulous individuals, and their abundance may increase after denture insertion (27). Other studies have shown that microbial loads vary among edentulous oral surfaces, with the dorsum of the tongue frequently carrying a greater burden than other mucosal sites (31). In addition, the same clonal type of *P. gingivalis* was identified over several years in an edentulous individual (32). These observations demonstrate that tooth loss does not necessarily eliminate *P. gingivalis* and that the organism may persist through prolonged colonisation of alternative oral niches. However, long-term detection or colonisation does not establish a persister phenotype, which requires demonstration of reversible physiological and antibiotic tolerance. Experimental studies have shown that metronidazole-surviving *P. gingivalis* persister populations can retain cellular morphology and the capacity to adhere to and invade host epithelial cells and to alter epithelial cytokine responses (33) indicating that persistent *P. gingivalis* colonisation could therefore represent a residual microbial reservoir with potential local or systemic relevance, but its contribution to cardiovascular risk after tooth loss remains hypothetical. Thus, evidence of persistent colonisation provides a clinical basis for considering mechanisms that could support prolonged bacterial survival in edentulous niches. Experimental studies showing that metronidazole-surviving *P. gingivalis* persisters retain epithelial adhesion, invasion, and immunomodulatory activity make persister cells a biologically plausible contributor, although their presence has not yet been directly demonstrated in edentulous patients. Moreover, associations between edentulism and cardiovascular outcomes are potentially influenced by prior periodontal inflammatory burden, age, smoking, diabetes, metabolic disease, socioeconomic factors, diet, frailty, and established cardiovascular disease.

Independently of the persister hypothesis, *P. gingivalis* peptidylarginine deiminase (PPAD) is located on the bacterial outer membrane and within OMVs and has been implicated in citrullination, biofilm organisation, polymicrobial interactions, virulence regulation, and host-immune disruption (34). Whether PPAD expression or activity differs between persister and non-persister populations has not been established.

### 3.2 The outer membrane vesicles (OMVs) as a possible pathway from persistent *P. gingivalis* reservoirs

Outer membrane vesicles are nanoscale, membrane-bound structures released by Gram-negative bacteria, including *Porphyromonas gingivalis* that can transport bacterial lipids, proteins, nucleic acids, enzymes, toxins, and other signalling molecules (35–38). In periodontal disease, *P. gingivalis*-derived OMVs can deliver concentrated virulence factors, interact with epithelial and immune cells, stimulate inflammatory responses, contribute to immune evasion, and support biofilm stability (35, 36). However, the direct evidence that *P. gingivalis* persister cells release OMVs is currently lacking. Also, OMVs derived from residual *P. gingivalis* populations have not been detected or quantified in edentulous patients, and their contribution to systemic vascular signalling after tooth loss has not been demonstrated. Therefore, the present model does not claim a proven persister-specific OMV pathway, but proposes the biologically plausible hypothesis that any metabolically active *P. gingivalis* population maintained within edentulous soft-tissue or denture-associated biofilms could potentially release OMVs as persister forms have been shown to retain bacterial morphology, adhere to host-cell surfaces, invade gingival epithelial cells, and alter cytokine responses in a manner similar to non-persister bacteria (33). If persister cells coexist with actively growing bacteria, the latter may represent the principal source of vesicles. Alternatively, persister cells could contribute indirectly by supporting recolonisation after stress removal rather than by producing OMVs during dormancy. Gram-negative bacteria commonly produce OMVs, and OMV production has been observed in biofilms and host-associated environments (38). Furthermore, OMV production is shaped by environmental conditions, including nutrient availability, oxidative stress, antibiotic exposure, and biofilm-associated stress (37–39) while OMVs may support bacterial survival by promoting virulence-factor delivery, immune modulation, biofilm stability, interbacterial communication, ecological adaptation, and antimicrobial defence (36, 38, 39). In edentulous oral niches, denture-associated and soft-tissue biofilms may create stress conditions that favour antibiotic-tolerant persister populations that are enriched in stationary-phase cultures and biofilms (40) and which may coexist with stress-regulated OMV production. Taken together, OMV-mediated signalling provides a plausible route by which persistent *P. gingivalis* populations could remain biologically relevant in edentulous oral niches. Even if persister-like cells are slow-growing or metabolically quiescent, low-level OMV-associated signaling may allow residual microbial reservoirs to influence local immune regulation, tissue stress, and systemic vascular biology after the removal of tooth-associated periodontal pockets.

### 3.3 *P. gingivalis* persisters alter the local microenvironment

A further hypothesis is that prolonged *P. gingivalis* colonisation may influence the local immune microenvironment. Experimental findings from metronidazole-treated *P. gingivalis* persisters provide one possible mechanistic example, but their applicability to edentulous oral niches remains untested. Namely, this evidence derives from a single *in vitro* model using human gingival epithelial cells and therefore cannot establish that the same cytokine response occurs *in vivo*, in edentulous mucosa, or under clinically relevant antimicrobial exposure. Metronidazole-treated *P. gingivalis* persisters downregulated 10 cytokines, including IL-6, IL-8, and CXCL5, with IL-6 among the validated suppressed mediators (33). This is immunologically relevant because IL-6 signaling in dendritic cell–lymphocyte crosstalk promotes effector T-cell proliferation and activation, particularly Th2 and Th17 responses, while inhibiting differentiation and activity of regulatory T cells (Tregs) (41). In an experimental persister model, reduced IL-6 signalling could theoretically shift CD4+ T-cell differentiation towards a more regulatory phenotype- Tregs rather than pro-inflammatory Th1 or Th17 cells. Whether a comparable cytokine pattern occurs in the edentulous mucosa has not been determined.

Although persisters are non-dividing or slow-growing, recent work shows that they maintain low-level but essential metabolic functions required for stress survival. Namely, nutrient alterations involving alternative carbon sources and metabolic intermediates, such as acetate, pyruvate, malate, succinate, fatty acid metabolism, glyoxylate, and amino-acid-related central metabolites, have been linked to antibiotic tolerance and persister-like states (29). A low-glucose, oxidative, or otherwise stressed microenvironment favour Treg survival relative to glycolysis-dependent Th17 responses (42). At present, the proposition of stressed oral microenvironment favouring Tregs is based on general immune-metabolic principles and has not been directly linked to *P. gingivalis* persisters in edentulous individuals. Th17 cells are highly dependent on glycolysis, show high GLUT1 expression, require substantial glucose for effector function, and are sensitive to glucose restriction and oxidative stress (43). In contrast, Tregs are less dependent on glycolysis, rely more on oxidative phosphorylation and fatty acid oxidation, and are better adapted to low-glucose or stress-associated environments (44). Clinically, the patient may lack signs of oral inflammation, while still have repeated low-grade microbial or vesicle-mediated signalling which can contribute to endothelial stress, immune modulation, and vascular inflammation.

## 4 Discussion

The present model proposes that persistent oral colonisation, local ecological stress, and host immune regulation may interact in edentulous niches. Experimental *P. gingivalis* persister studies provide a possible mechanism through altered epithelial cytokine responses, but direct evidence for persister cells, persister-driven Treg dominance, or persister-derived OMVs in edentulous patients is lacking. The cytokine and metabolic changes induced by *P. gingivalis* persisters may favour a Treg-dominant, immunologically tolerant oral environment, weakening bacterial clearance and supporting chronic colonisation despite the absence of overt periodontal inflammation (33, 41) (Figure 2). In the proposed model, these residual microbial communities may influence the cardiovascular system through several mechanisms: episodic translocation of bacteria or bacterial components across denture-traumatized or otherwise compromised mucosa; release of virulence factors and outer membrane vesicles capable of affecting epithelial, immune, and potentially endothelial signalling; and maintenance of low-grade systemic immune and metabolic stimulation resulting from incomplete microbial clearance.

**Figure 2:**
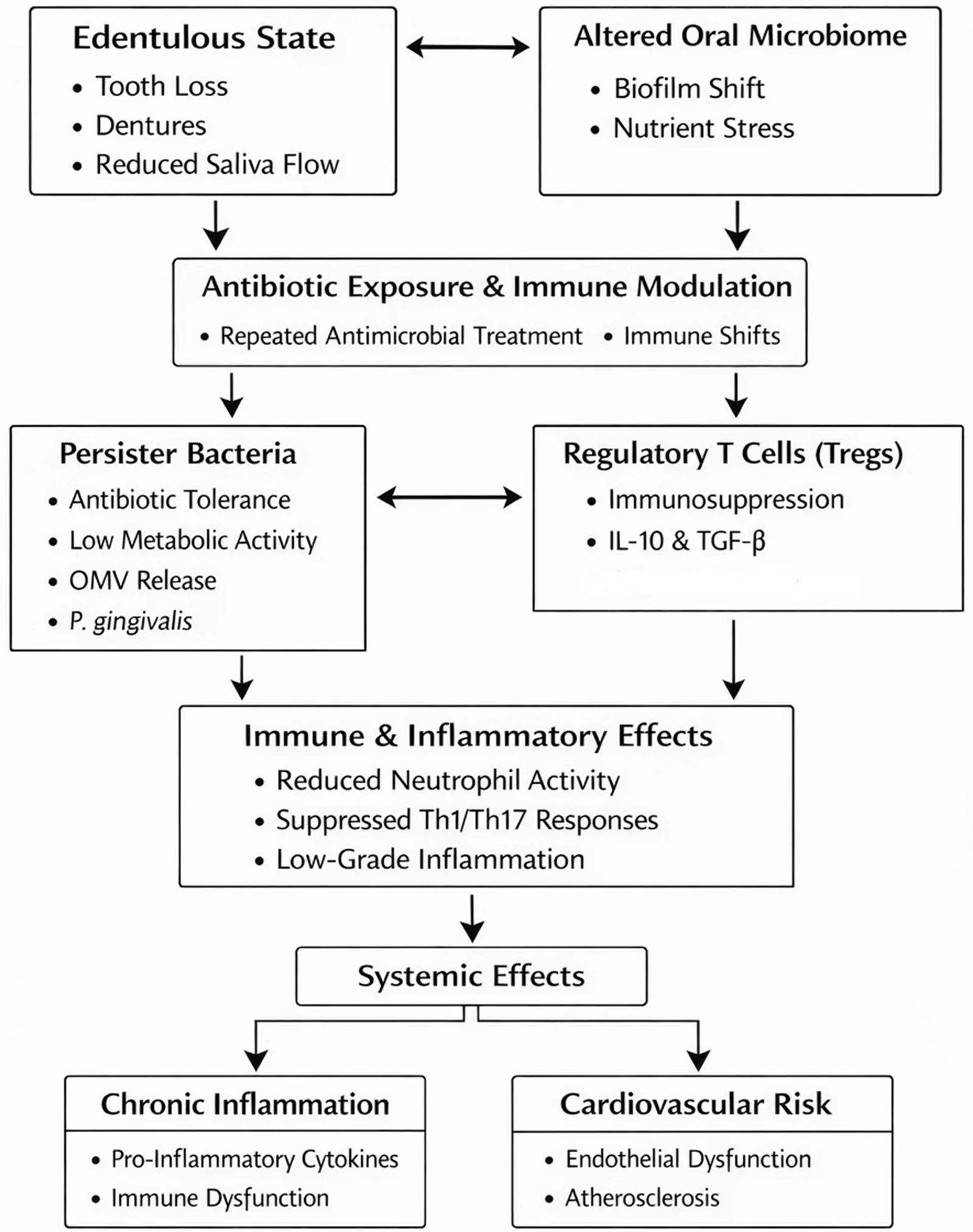
Hypothesis-generating model linking edentulous oral environment with residual cardiovascular risk. Tooth loss removes periodontal pockets but creates a reorganised oral environment influenced by mucosal surfaces, denture use, salivary changes, nutrient availability, microtrauma, and hygiene. These conditions may permit persistent colonisation by *P. gingivalis* and other organisms in alternative oral niches. A phenotypically antibiotic-tolerant persister subpopulation is one possible contributor to prolonged bacterial survival, but direct evidence for *P. gingivalis* persister cells in edentulous patients is lacking. These metabolically quiescent but stress-tolerant bacteria may persist in alternative oral niches and contribute to host modulation through immune-suppressive signalling and outer membrane vesicle (OMV) release. In parallel, the altered low-glucose, stress-associated microenvironment may preferentially support regulatory T cells (Tregs), which rely more on oxidative phosphorylation and fatty acid oxidation than on glycolysis. Hypothetically, Tregs may suppress local antimicrobial immunity through several mechanisms, including the production of IL-10 and TGF-*β*, inhibition of effector T-cell responses, and reduced neutrophil-recruiting signals thereby weakening local bacterial clearance and promoting further persistence of *P. gingivalis*. Together, persistent colonization, OMV-mediated signalling, and Treg-dominant immune tolerance may sustain low-grade systemic inflammation and tissue stress despite the absence of periodontal infection, providing a plausible pathway through which cardiovascular risk may persist after tooth loss.

This interpretation is biologically plausible because Tregs are commonly increased in chronic infections and mucosal tissues, including the oral cavity (45). In edentulous patients, low-grade irritation from dentures, microtrauma, or residual biofilm may further support a Treg-favouring environment (45). Edentulism has also been associated with uncontrolled hypertension and altered IL-2 levels, suggesting a possible link between tooth loss, immune regulation, and vascular risk (46). Noteworthy, IL-2 is required for Foxp3 expression, Treg differentiation, and maintenance of suppressive Treg activity (47, 48). In addition, plasma HDL cholesterol has been positively associated with circulating Treg subsets, further supporting a possible connection between adaptive immune regulation and metabolic cardiovascular markers (49). Locally, Treg dominance may weaken antimicrobial defence by suppressing Th1-, Th17-, and neutrophil-mediated responses (41) that are important for elimination of *P. gingivalis* and allow biofilm-associated or persister-like *P. gingivalis* to survive longer and evade immune clearance.

The Treg-driven tolerance may also extend beyond the oral cavity through systemic metabolic and immune effects. Rather than acting through classical inflammatory injury from active periodontal pockets alone, edentulism-related cardiovascular risk may involve a fine interaction between immune tolerance, microbial persistence, and host metabolic stress. In patients with stable coronary heart disease, Vedin et al. (50) showed that tooth loss was independently associated with higher levels of several prognostic biomarkers, including growth differentiation factor 15 (GDF-15), a stress-responsive cytokine, and N-terminal pro-B-type natriuretic peptide (NT-proBNP), a marker of cardiac wall stress, both associated with cardiovascular risk and immune regulation (51, 52). These associations do not prove causality, but they suggest that edentulism-related cardiovascular risk may involve pathways beyond classical atherosclerotic inflammation alone.

Future studies are needed to test this hypothesis more directly. Firstly, the direct evidence on how residual oral microbial reservoirs influence cardiovascular biology in edentulous individuals is lacking. The gastrointestinal tract may also act as an intermediate component of an oral–gut–vascular axis, because swallowed oral microorganisms and their products could alter the intestinal microbiota, epithelial barrier function, and systemic exposure to microbial mediators (53). However, this pathway has not been demonstrated specifically in edentulous patients. Although denture cleansers may reduce denture-associated microbial burden (54), they are unlikely to eliminate biofilms completely because dentures can be recolonised from the tongue, saliva, and oral mucosal surfaces, while their effects on persister populations and systemic cardiovascular biomarkers remain unknown. Longitudinal and interventional studies combining oral and gut microbiome profiling, detection of *P. gingivalis* DNA or outer membrane vesicles, assessment of mucosal and circulating immune responses, and evaluation of denture-hygiene interventions are therefore required to establish whether these pathways contribute causally to persistent cardiovascular risk after tooth loss. Moreover, the proposed microbial–immune pathway is only one possible explanation for persistent cardiovascular risk after tooth loss, which may also reflect prior inflammatory exposure, shared genetic and metabolic risk, smoking, diabetes, socioeconomic factors, dietary changes, frailty, or progression of established vascular disease. These mechanisms may act independently or together, so the present model should be regarded as an additional, testable pathway rather than a replacement for established explanations.

In conclusion, persistent cardiovascular risk in edentulism may partly involve continued colonisation of alternative oral niches by periodontal organisms and altered local immune regulation. A *P. gingivalis* persister subpopulation, Treg-favouring immune conditions, and OMV-mediated signalling represent biologically plausible but currently unconfirmed components of this pathway. This model provides a new perspective on oral-systemic interactions after tooth loss and suggests a basis for future targeted investigation and intervention.

## Data Availability

The original contributions presented in the study are included in the article/Supplementary Material, further inquiries can be directed to the corresponding author.
